# Microscopic-scale magnetic recording of brain neuronal electrical activity using a diamond quantum sensor

**DOI:** 10.1038/s41598-023-39539-y

**Published:** 2023-07-31

**Authors:** Nikolaj Winther Hansen, James Luke Webb, Luca Troise, Christoffer Olsson, Leo Tomasevic, Ovidiu Brinza, Jocelyn Achard, Robert Staacke, Michael Kieschnick, Jan Meijer, Axel Thielscher, Hartwig Roman Siebner, Kirstine Berg-Sørensen, Jean-François Perrier, Alexander Huck, Ulrik Lund Andersen

**Affiliations:** 1 Department of Neuroscience, University of Copenhagen, 2200 Copenhagen, Denmark; 2 Center for Macroscopic Quantum States (bigQ), Department of Physics, Technical University of Denmark, 2800 Kongens Lyngby, Denmark; 3 Department of Health Technology, Technical University of Denmark, 2800 Kongens Lyngby, Denmark; 4 Danish Research Center for Magnetic Resonance, Center for Functional and Diagnostic Imaging and Research, Copenhagen University Hospital - Amager and Hvidovre, 2650 Hvidovre, Denmark; 5 Laboratoire des Sciences des Procédés et des Matériaux, Université Sorbonne Paris Nord, 93430 Villetaneuse, France; 6 Division Applied Quantum System, Felix Bloch Institute for Solid State Physics, Leipzig University, 04103 Leipzig, Germany; 7 Department of Neurology, Copenhagen University Hospital Bispebjerg and Frederiksberg, 2400 Copenhagen, Denmark; 8 Department of Clinical Medicine, Faculty of Health and Medical Sciences, University of Copenhagen, 2200 Copenhagen N, Denmark

**Keywords:** Optics and photonics, Physics

## Abstract

Quantum sensors using solid state qubits have demonstrated outstanding sensitivity, beyond that possible using classical devices. In particular, those based on colour centres in diamond have demonstrated high sensitivity to magnetic field through exploiting the field-dependent emission of fluorescence under coherent control using microwaves. Given the highly biocompatible nature of diamond, sensing from biological samples is a key interdisciplinary application. In particular, the microscopic-scale study of living systems can be possible through recording of temperature and biomagnetic field. In this work, we use such a quantum sensor to demonstrate such microscopic-scale recording of electrical activity from neurons in fragile living brain tissue. By recording weak magnetic field induced by ionic currents in mouse *corpus callosum* axons, we accurately recover signals from neuronal action potential propagation while demonstrating *in situ* pharmacology. Our sensor allows recording of the electrical activity in neural circuits, disruption of which can shed light on the mechanisms of disease emergence. Unlike existing techniques for recording activity, which can require potentially damaging direct interaction, our sensing is entirely passive and remote from the sample. Our results open a promising new avenue for the microscopic recording of neuronal signals, offering the eventual prospect of microscopic imaging of electrical activity in the living mammalian brain.

## Introduction

Neurodegenerative diseases in the brain are characterised by substantial microscopic-scale structural changes, including amyloid-$\beta$ (A$\beta$) and tau tangles aggregation^1,2^ or inflammation in the case of multiple sclerosis (MS)^3^, which can occur well before disease becomes symptomatic^4,5^. This includes microscopic physical structural changes associated with alterations of action potential propagation^2,6^. Studying small-scale functional changes in the very early stages of degeneration is of utmost importance in understanding the mechanisms of disease emergence and for the eventual development of early intervention treatment. A key tool towards this goal is the study of the early onset of disease induced in an animal model^1,7,8^. Using dissected tissue, such studies of early disease onset are currently achieved by techniques such as patch clamp or multi-electrode arrays, or by laser/LED light combined with the introduction of voltage-sensitive dyes or the expression of genetically encoded voltage imaging^9^. Depending on the type of electrodes used, the former technique can be invasive and the data from experiments dependent on the setup and sample conditions, such as the quality of electrical contact or mechanical wear of the underlying or insertable electrodes. The latter technique can depend on localised optical properties of the surrounding tissue^10^, can demand genetic engineering and can have detrimental phototoxicity from high intensity laser light and cell toxicity due to the voltage sensitive dyes used^9^. Furthermore, both techniques can be specific to the biology of the target tissue, requiring adaptations between different tissues or structures. For example, Ca$^{2+}$ imaging of axonal axial current can be challenging due to the low number of Ca ions, posing challenges for studies of neurodegenerative diseases (e.g. MS) involving such structures^3^.

A technique that allows repeatable, passive, microscopic-scale recording of electrical activity in any kind of dissected living tissue without these disadvantages is therefore desirable. One such method is to record the biomagnetic field induced by ionic currents associated with action potentials in neurons. Although such fields are extremely weak (nano- to femto-Tesla), magnetic field can freely permeate biological tissue with minimal interaction, avoiding damage and signal distortion and allowing remote recording. For low resolution sensing in whole human and animal subjects, magnetic field sensing has been implemented successfully as magnetoencephalography, giving centimetre-scale imaging of brain electrical activity from outside the body. However, this methodology relies on superconducting quantum interference devices (SQUIDs)^11^ or more recently spin-exchange relaxation-free (SERF)^12^ sensors, respectively demanding cryogenic cooling or a high temperature atomic vapour. Alongside other disadvantages, the intrinsically non-biocompatible properties of these sensors mean that they cannot easily be brought into sufficiently close proximity (tens of micrometers) to the target sample necessary for microscopic-scale recording, particularly where a solution bath is required to keep tissue alive.

What is therefore required is a new type of biocompatible sensor, capable of microscopically resolving and imaging electrical activity in dissected tissue *in vitro*. In the past years, an alternative has emerged in the form of colour centres, optically active point defects in solid state materials^13^. Colour centres, in particular nitrogen-vacancy centres in diamond, have been employed as a new generation of quantum sensor^14^ to detect biomagnetic field induced by electrical activity^15,16^, offering remote and passive microscopic-scale recording of electrical activity based purely on fundamental physical principles, independent of the biological target system, in a highly biocompatible host material. Sensing operates via the principle of optically detected magnetic resonance (ODMR) spectroscopy^17^, where biomagnetic field modifies colour centre fluorescence emission under illumination with control fields (microwaves and laser light). Absolute magnetic field sensitivity can be maximised by using a large ensemble of colour centres and strong control fields in high proximity to the biological sample. However, this setup with strong laser light and associated heating of the host material and strong microwave fields (potentiality driving water resonance) can be highly damaging to fragile biological systems. This has limited the applicability of colour centre-based microscopic sensing to more robust biological samples, rather than the fragile mammalian tissue most of interest for animal models of human disease.

In this work, we overcome these difficulties in a proof of principle experiment to demonstrate the first microscopic-scale recording of action potential propagation in tissue from fragile mammalian brain tissue using biomagnetic field recording. We use a quantum sensor based on nitrogen-vacancy colour centres in diamond to record magnetic field from axial current associated with action potentials propagating in axons in the *corpus callosum*, which connects the two cerebral hemispheres and enables integration of sensory-, motor- and cognitive information. This structure is very suitable for biomagnetic recording, having a high density of axons in a microscopic-scale region and a well-studied, strong and quantifiable biological response. Furthermore, the corpus callosum is of interest as small-scale functional changes and atrophy can be early indicators of neurodegenerative disease^18–21^.

Using a tissue slice dissected from the brain of a mouse, with the slice kept alive in vitro in a carbogenated solution bath, we record magnetic field from the action potentials propagating in callosal axons within a microscopic sensing region. Recording is performed passively and remotely, with the slice physically separated from the magnetic field sensor and with no need to direct any laser light into the slice. We recover biomagnetic signals with distinct features that correspond to action potentials from both myelinated and unmyelinated callosal axons. We verify our biomagnetic recordings by simultaneous electrophysiological measurements using an invasive probe electrode inserted into the *corpus callosum*. Finally, we demonstrate the capability to perform in situ pharmacology on the tissue while recording, of importance for the study of target biosystem dynamics and for eventual evaluation of effectiveness of prospective drugs against disease. This is achieved by using a sodium channel blocker tetrodotoxin as an inhibitor, modifying action potential propagation^22^.

### Biomagnetic recording of compound action potential

**Figure 1:**
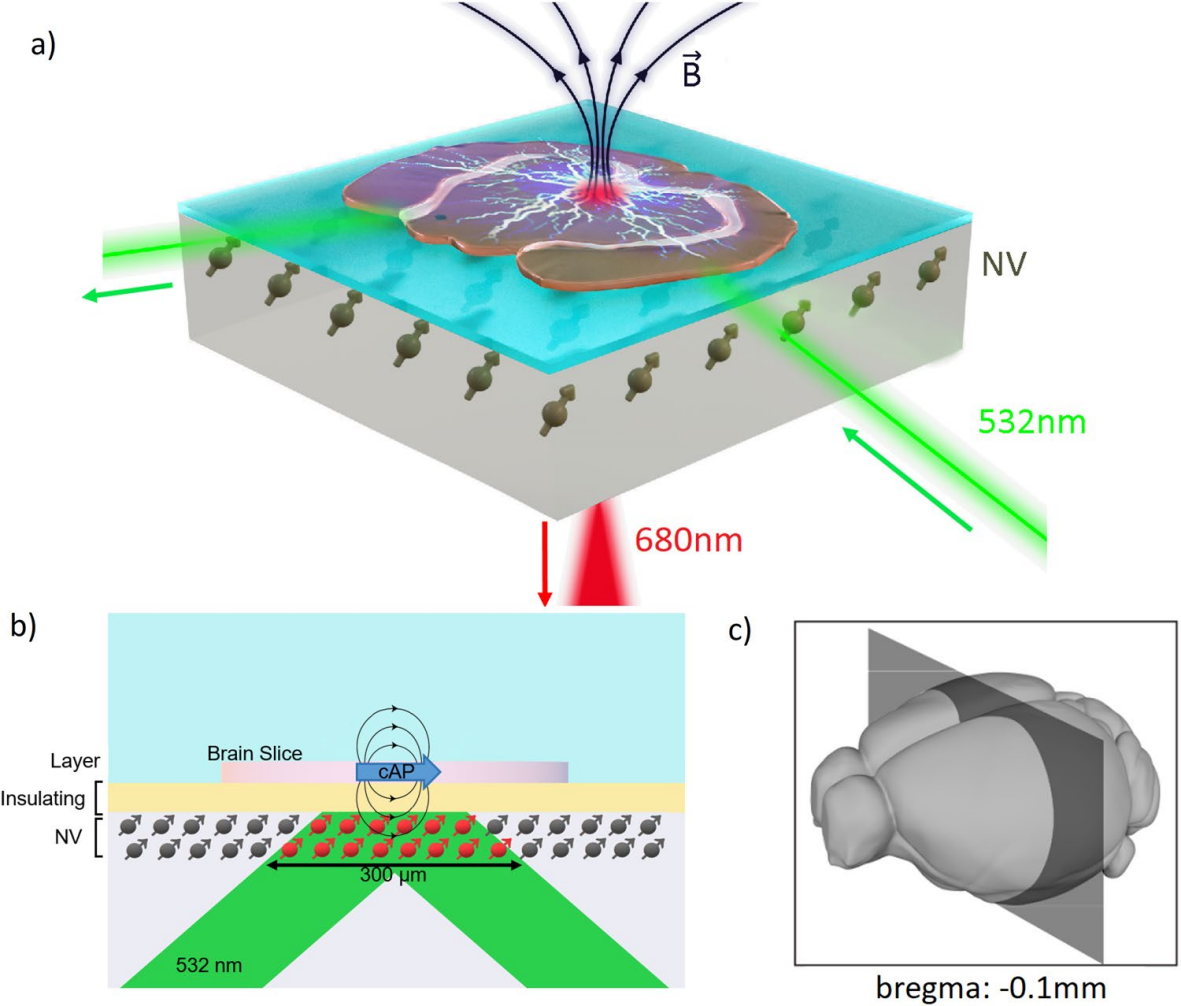
Sensor principle of operation and slice location in the brain. (**a**) Schematic of the sensor operation (not to scale), where green laser light directed to subsurface colour centres (NV) in the diamond enables recording of magnetic field arising from compound action potentials (cAP) in a brain tissue slice placed above the diamond. (**b**) Side-on schematic, detailing the operating principle with colour centres illuminated by the 532nm pump laser recording the field induced by cAP-associated current. The laser is reflected by thin Al foil under a Kapton insulating layer and does not pass through the slice. (**c**) 3D diagram of the mouse brain, showing the location of the slice used in experiments.

**Figure 2:**
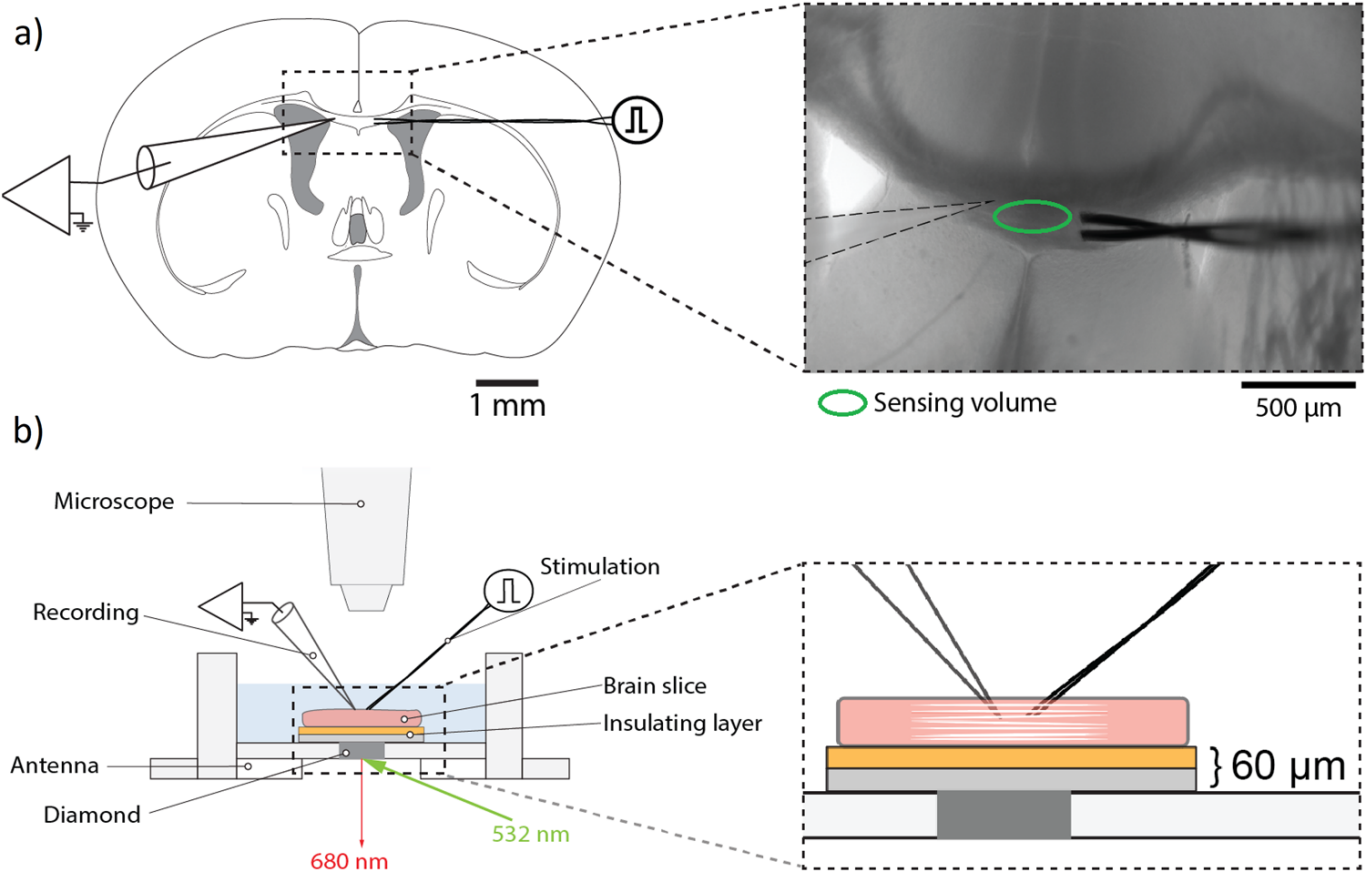
Slice preparation and experimental schematic. (**a**) Top-down illustration and image of a coronal brain slice from a mouse. The stimulation and invasive electrical recording electrodes are positioned in the *corpus callosum* and the dark grey square indicates the position of the diamond beneath the slice, with the active sensing region marked. (**b**) Side-on schematic of the recording chamber, with magnified central region.

The operating principle of our sensor is illustrated schematically in Fig. 1a and b with further details given in Methods. Using ODMR spectroscopy^23^, magnetic field induced frequency shifts in the microwave spin resonances of the nitrogen-vacancy colour centres are transduced to changes in emitted fluorescence intensity $I_{FL}$ when pumped with 532nm laser light and low power microwaves. Changing fluorescence intensity $\Delta I_{FL}(t)$ can be recorded by a photosensitive detector and translated to a time dependent recording of absolute magnetic field strength observed at the diamond $\Delta I_{FL}(t)$ $\propto$ $\gamma _e$ $B(t)$, using the gyromagnetic ratio of the nitrogen-vacancy electronic spin $\gamma _e$ = 2$\pi$ $\times$28Hz/nT. Each spatially separate defect in a high density layer of centres close to the surface of the host diamond material can act as an independent sensor, allowing nanometer-scale resolution of magnetic field^24^. The colour centres are sub-surface, protected within the host material, yet close enough to the surface to accurately sense nearby magnetic field, making the sensor robust and biocompatible. They are also strong, stable optical emitters, which enables high magnetic field sensitivity with wide sensing bandwidth. Here we achieve sensitivity of 50pT/$\sqrt{Hz}$ with a sensing bandwidth of $f_{-3dB}$=10kHz using a 300$\times$100$\times$20$\mu$m$^3$ volume of colour centres addressed by the pump laser. We include a plot of the spectral response of our sensor in Supplementary Information. We highlight that the pump laser light is entirely contained within the diamond, and does not pass through or in any way directly interact with the solution bath or tissue under study.

In Fig. 2a we show a schematic and white light image of our slice preparation (direction indicated in Fig. 1c including electrode locations in the *corpus callosum*. In Fig. 2b we schematically show how the slice is introduced into our recording chamber, containing a bath of chilled, carbogenated artificial cerebrospinal fluid (ACSF) located above the sensor. The position of the slice could be freely manipulated in the chamber, with placement determined using a white light microscope mounted above, such that the *corpus callosum* could be precisely located in the approximately 300$\times$100$\mu$m$^2$ sensing area above the diamond. The slice was separated from the diamond by an optically and electrically insulating layer, with sample-sensor distance approximately 60$\mu$m. Bath temperature above the diamond could be maintained at a stable 25C. Electrical activity in the *corpus callosum* was induced by a Pt/Ir bipolar stimulation electrode located 1-2 mm away from the sensing region, generating a compound action potential and associated ionic currents in callosal axons. For direct comparison to our biomagnetic recordings, compound action potentials were simultaneously recorded by measuring the local field potential with an invasive AgCL coated silver electrode positioned on the contralateral side, closely adjacent to the location of biomagnetic sensing. We detail measurements from a limited number (n=3) of slices to provide proof-of-principle demonstration of our sensor; we do not seek in this work to perform statistical analysis of the observed biological response.

**Figure 3:**
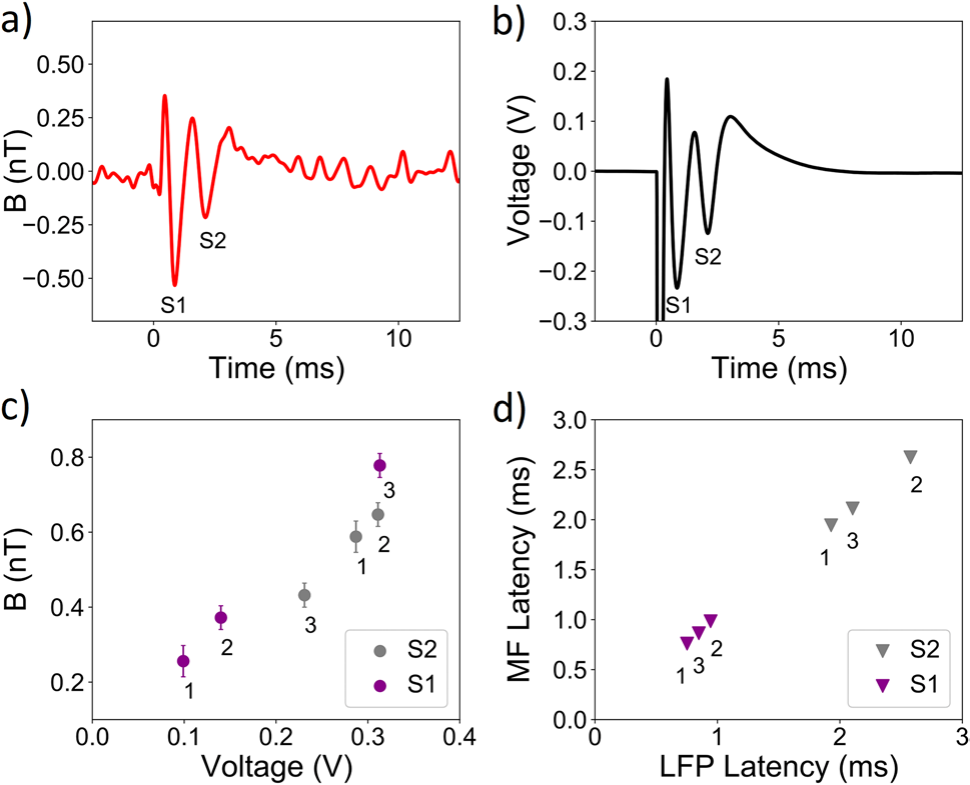
Detection of compound action potential from the corpus callosum. (**a**) Biomagnetic signal recorded by the quantum sensor from the *corpus callosum*, displayed with a bandwidth of 2.5kHz after filtering. (**b**) Simultaneous local field potential (LFP) recording of the *corpus callosum* compound action potential from the same region. Here, the tissue was stimulated electrically at *t*=0ms. Features S1 and S2 correspond to action potential from myelinated and unmyelinated axons respectively. (**c**) Amplitude of S1 and S2 as recorded by invasive electrical probe versus their amplitude from the biomagnetic recording. Here data is shown from 3 slices taken from different mice. The strength of the biomagnetic signal was linearly proportional to the amplitude of the local field potential. (**d**) The same proportionality was also observed for latency, here shown for components S1 and S2 for invasive probe and biomagnetic recordings from the same three slices.

We recorded a total of 28,800 trials at a stimulation frequency of 2 Hz over a period of 4 hours from each individual slice. We recovered a detectable (SNR>5) biomagnetic signal when averaged over a minimum of 300 trials ($\approx 3 mins$). Although single-trial readout would be preferable, our sensitivity is as yet insufficient to reach this level of performance. Figure 3a shows the simultaneously recovered electrophysiological signal of compound action potential propagation in the *corpus callosum* and Figure 3b the biomagnetic signal, recorded and averaged over 60 minutes. We recovered a biomagnetic signal from our quantum sensor with the same characteristic features as the local field potential signal recorded electrically by the invasive probe. Our signal arises from basic physical principles for the magnetic field induced by a flow of electrical current (Ampere’s circuital law). We observed two distinct features in both datasets, labelled S1 and S2 in Fig. 3. Features S1 and S2 are understood in the literature to arise from compound action propagation primarily in myelinated and unmyelinated axons respectively^25^, with some continuum of overlap (i.e. a minor myelinated contribution to S2 and *vice versa*). The relative amplitude and latency characteristics were the same for both S1 and S2 in both biomagnetic and electrical probe data. S1 and S2 in the biomagnetic readout changed in amplitude and latency in linear proportion to the equivalent local field potential, with a stronger electrical response corresponding to a stronger biomagnetic field signal. This proportionality was consistent when the experiment was repeated for multiple slices from different mice, as shown in Fig. 3c and d. We note that the ratio between amplitudes of S1 and S2 can vary depending on the ratio of myelinated to unmyelinated axons in the corpus callosum, which can vary significantly between individual animals^26^. An advantage of biomagnetic recording was the minimisation of the strong stimulation artifact electrically induced in the local field potential recording, offering the benefit of recovery of signal features much closer to the stimulation time ($t$ 0.5ms). Further detail of how this was achieved is described in Supplementary Information.

### *In-situ* pharmacology with tetrodotoxin

**Figure 4:**
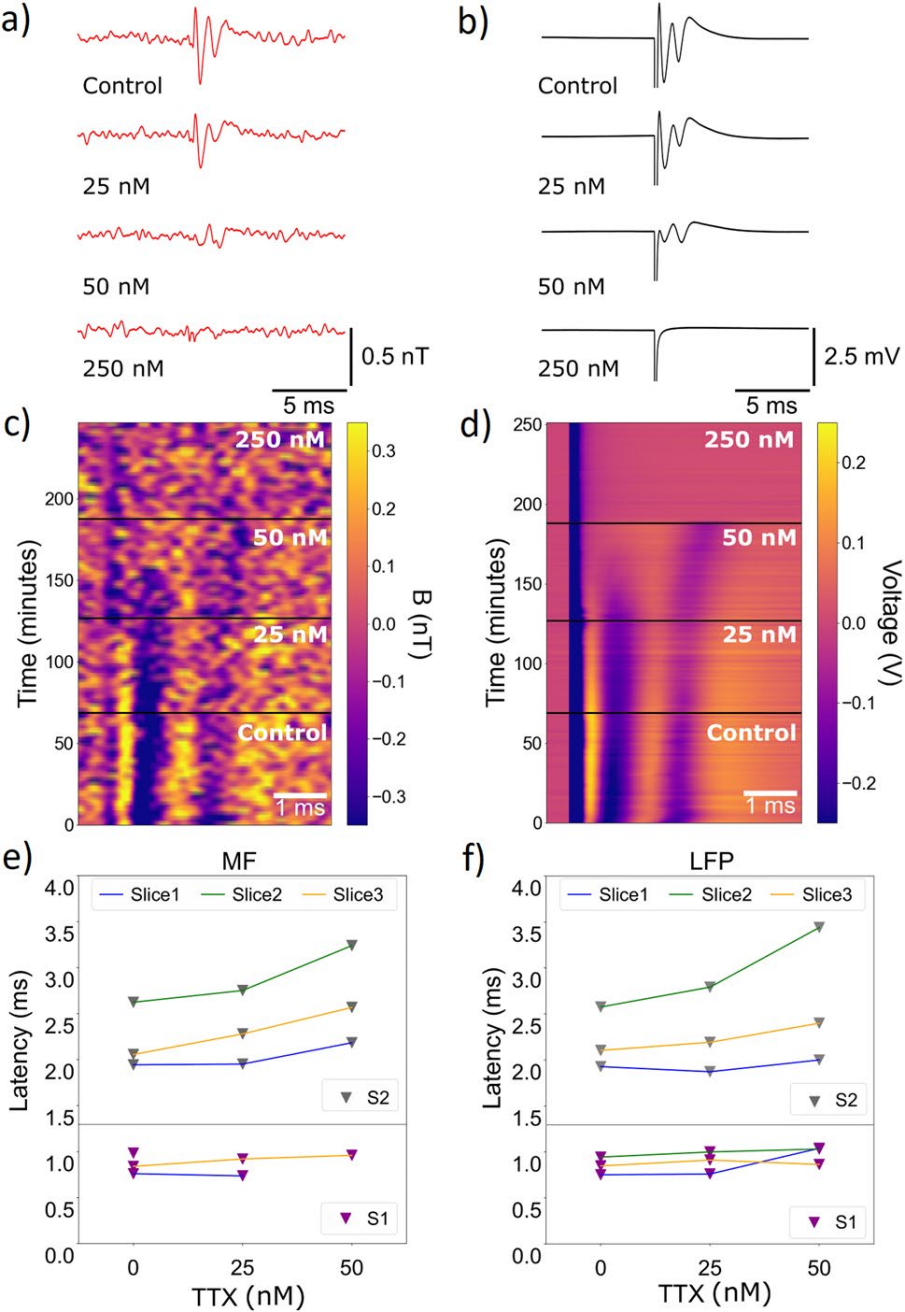
The effect of the introduction of tetrodotoxin on compound action potential propagation in the corpus callosum. (**a,b**) Local field potential (LFP, black) measure by invasive electrical probe and simultaneous biomagnetic signal from the quantum sensor (red) recorded from the *corpus callosum* as a function of increase in tetrodotoxin concentration over the 4 hour duration of the experiment. (**c,d**) Color plots of the LFP and quantum sensor recording as the experiment progressed. Each y-axis step represents the average of 360 trials (3 minutes of recording). In B and C, S1 and S2 components appear in purple. (**e,f**) Quantification of the latency shift in the LFP and biomagnetic field signal respectively caused by tetrodotoxin for S1 (bottom) and S2 (top) for 3 tissue slices from different mice.

To determine whether our sensor could detect subtle changes in axon action potential waveform, comparable to what happens in the course of neurodegenerative diseases^27^, and to illustrate our capability to perform simultaneous pharmacology during recording, we inhibited the action potential signal by blocking voltage sensitive sodium ion channels using tetrodotoxin^28^. This was added in increasing nanomolar concentration to the solution bath containing the brain tissue slice while recording from the *corpus callosum* using the quantum sensor and again simultaneously with an invasive electrical probe. The resulting effect of increasing tetrodotoxin concentration on the electrical signal can be seen in Fig. 4b and d with the comparative effect on the biomagnetic signal in Fig. 4a and c. After a 60 min control recording, concentration was increased to 25nM, then to 50nM after a subsequent 60min recording period, with final large concentration of 250nM added after 3 hours of recording, to fully inhibit the signal. We include in Supplementary Information the equivalent data plots for a control slice with no introduction of tetrodotoxin.

In the magnetic data recorded by our quantum sensor and the simultaneous electrical recording, we observed the reduction in amplitude and elimination of the fast S1 signal feature and the increase in latency of the slower S2 feature, up to and including concentrations of 50nM. The reduction in S1 and S2 amplitude has been previously established to arise through decrease in the number of functional sodium ion channels. Myelinated axons have a high proportion of more TTX-sensitive Nav$_{1.6}$ sodium channels compared to unmyelinated axons which mainly express the less-TTX sensitive Nav$_{1.2}$ sodium channels, making myelinated axons more susceptible to tetrodotoxin^29–31^. This was specifically observed as a faster decrease in amplitude of feature S1 with increasing levels of tetrodotoxin compared to S2. The blocking of sodium channels was also observed as a decrease in the conduction velocity of unmyelinated axons, seen as an increase in the latency of signal S2 with higher concentration^22^. These behaviours were again observed consistently in both magnetic and electric recordings and between tissue slices from different mice as shown in Fig. 4e and f. In the presence of high tetrodotoxin concentration (250 nM), the biological signal was abolished by blocking the majority of sodium ion channels in both myelinated and unmyelinated axons, in order to verify our recording of action potential rather than artifact signal.

In summary, we have successfully demonstrated the recording of neural activity from living brain tissue at the microscopic scale using a diamond quantum sensor. As our technique directly records magnetic field, no modification of the target system biology is required, and therefore the technique is able to record any ionic current from any region within any type of tissue section. The technique is sensitive enough and has sufficient time resolution to detect subtle changes in electrical activity. This we demonstrate in a specific target region of the brain, axons in the *corpus callosum*, recording the biomagnetic field from action potentials arising from axons of different types (myelinated and unmyelinated), with good agreement with simultaneous control measurements using an electrical probe. Our technique can enable detailed microscopic study of pathologies affecting neuronal electrical activity in animal disease models.

We highlight that the measurements we present in this work are substantially more challenging than previous work on much more biologically robust living systems such as marine invertebrates^32^ or cardiac tissue in living whole-animal study^33^. The first major difficulty is that the LFP signals from mammalian brain tissue ($\approx$1–4 mV) are weaker than those generated in muscle tissue ($\approx$10–20 mV). This can be attributed to the smaller size of the brain axons and significantly higher transmembrane current generated by individual muscle fibers, typically being one or two orders of magnitude stronger than that of axons. Furthermore, the magnitude of LFP signals in brain slices is heavily influenced by the relative conduction velocity and spatial organization of axons. In brain regions where axons exhibit similar diameters and run in parallel directions, such as the hippocampus, strong LFP signals can be reliably evoked. Conversely, in regions with minimal organization among neurons, the recorded LFP signals are often negligible or nonexistent due to signal cancellation. In the case of the corpus callosum, where axons vary in diameter and thereby exhibit different conduction velocities, LFP amplitudes of around 1 mV are typically observed. These factors can all result in a far weaker recorded magnetic field, which can be further reduced by extracellular return currents and by the fact that the mammalian neurons may be embedded deep within tissue, rather than being near-surface, increasing source-sensor separation. In this work, the structure of the corpus callosum is relatively simple. However, we anticipate that issues with field cancellation may become significantly worse in even more complex neural structures, demanding higher spatial resolution.

The second major difficulty is that the tissue is extremely fragile, with only slight deviation from optimal conditions permanently ending all neuronal activity. Mammalian brain tissue can be easily damaged mechanically, requiring careful procedure design to place the tissue on the sensor in the sample bath. We also found it extremely sensitive to anoxia; it was necessary to carefully optimise bath design to ensure enough of a supply of fresh carbogenated solution to the tissue bath to avoid this problem. Finally, maintaining strong activity was extremely sensitive to deviations in local temperature, a problem which whole-animal *in vivo* study (maintaining homeostasis) and non-mammalian tissue is much more robust against. Our experimental design and methods maximised laser heat transfer away from both the diamond and the tissue while precisely controlling bath temperature. Finally, we found sensitivity to rapid switching of the microwave field, which we attribute to either direct action of the microwaves on the tissue or indirect action via water heating. Although we were able to solve the problem in this work by carefully and slowly ramping microwave power, this has negative implications for the prospect of future pulsed sensing experiments.

We further emphasise that although colour-centre quantum sensing and neuronal activity in the brain of the mouse are individually well studied, the novelty of this work comes in the cross-disciplinary approach, demonstrating the applicability of colour centre-based quantum sensing to weak magnetic field signal from a highly fragile biological system. Although our work represents a far earlier stage of technological development, it offers eventual prospective advantages over well-established microscopic techniques. As compared to existing optical methods, there is no need to direct laser light into the tissue, no need to modify the target biological system either genetically or via the introduction of potentially toxic fluorescent dyes and no reliance on the presence of specific ion types. Against electrical probes, there is no distortion by the variability in quality of electrical contact or relative conductivity of the surrounding tissue, no need to invasively insert point electrodes into the tissue and no deterioration of either sensor or sample on repeated sample exchange as the NVs record entirely passively, protected within the diamond. Although beyond the scope of this work, the technique offers the future prospect of high spatial imaging resolution by exploiting the high, consistent densities of colour centres^34,35^.

## Methods

### Diamond quantum sensor

Our sensor is based on a [100] oriented electronic-grade diamond crystal (Element Six). Diamond dimensions were 2 $\times$ 2 $\times$ 0.5 mm$^3$. The diamond was overgrown via chemical vapour deposition (CVD) to a thickness of approximately 20$\mu$m, forming a diamond layer with $\approx$5 ppm of $^{14}$N doping in the overgrowth. N to NV conversion was achieved by proton irradiation at 2.8MeV followed by annealing at 800 °C in an inert atmosphere. Sensing was performed using NVs formed by irradiation near the top surface in the CVD overgrown layer, with the remaining thickness (total 500$\mu$m) of the diamond substrate giving minimal contribution to field sensing.

Our ODMR resonance linewidth was 1 MHz and contrast of 1.5% for a single NV axis. For dissipation of heat generated by the pump laser, we mounted the diamond in a laser cut aluminium nitride heatsink. The top surface of the diamond was covered by 16$\mu$m thick aluminium foil, acting as an additional heatsink and reflecting pump laser back into the diamond. A layer of Kapton tape (50$\mu$m) above the foil electrically insulated the sample from the diamond. Microwave were supplied by a custom-designed printed board microwave antenna placed below the diamond and AlN heatsink. Our biological tissue was contained in a solution bath in a 3D printed plastic chamber, mounted directly above the diamond and made watertight using bio-safe silicone.

We optically pumped our NV centres with 1.2–1.4W of linearly polarised single mode 532nm green laser light (Coherent Verdi G2), coupled into the diamond at Brewster’s angle (67°) from beneath the diamond. No pump light passed through the solution bath or biological sample. The pump light was focused into the diamond using a f=400mm plano-convex lens (Thorlabs), striking the diamond top surface in a spot size of approximately 300×100$\mu$m. Assuming the majority of NVs are created by irradiation in the top 20$\mu$m (CVD overgrowth layer), we approximately estimate our sensing volume as the laser spot size times the overgrowth layer depth. This represents an upper limit for the region of NVs contributing to the magnetic field signal, as those nearest the surface and closest to the centre of the Gaussian beam (maximum laser power, optimal microwave power) may contribute to a greater degree to the measured contrast, change in fluorescence and hence magnetic field signal. We note that NV sensing volume could be reduced, increasing magnetic field spatial resolution, by tighter optical focus, using higher pump power to maintain sensitivity (valid while remaining below NV pump saturation levels).

NV centre fluorescence was collected by a 12mm condenser lens (Thorlabs ACL 1210) and the pump light removed by an optical longpass filter (FEL0600). Biomagnetic field was recorded via changes in intensity of red fluorescence emission using an electronically balanced optical receiver (Nirvana 2007, New Focus Inc.). Our overall magnetic sensitivity was constrained by the level of balanced detector common mode rejection (50dB) and primarily by the maximum level of fluorescence input signal into the photodetector (5-6 mW, depending on operating mode) before saturation of detector amplifiers. This limited pump power to <1.5W without additional attenuation of the fluorescence. Additional sensitivity, potentially to the shot noise limit, could otherwise be reached by increasing pump power (up to 5W).

We measured magnetic field response only in a single direction, defined by a static bias field of 1.5 mT applied parallel to the diamond [110] crystallographic direction. We used a continuous wave scheme with three-frequency microwave driving^36^ using two microwave generators (Stanford SG394) tuned to the spin transition (2.7–3GHz) and the $^{14}$N hyperfine transitions (2.16 MHz). The microwaves were modulated at 33.3 kHz for lock-in detection (Stanford SR850), with time constant of 10$\mu$s leading to a -3dB rolloff measurement bandwidth of $\approx$10kHz. Lock-in amplifier output was digitised at 125 kSa/s by an analog to digital converter (ADC, NI PCI-6221) and processed using custom Labview software.

Data was simultaneously acquired using a AgCL coated silver electrode, inserted into the corpus callosum in close proximity to the colour centre sensor. Readout was performed using a preamplifier stage and amplifier (Axon Instruments) and recorded by the same ADC as the magnetic data. To ensure no crosstalk between the electrical probe and the magnetic data, the amplified output voltage was minimised and decoupled from the colour centre sensor measurement electronics using an optocoupler. Tests were performed with the colour centre sensor microwave drive frequency set away from the NV centre resonance frequency (magnetically insensitive) while recording electrically from the slice; no crosstalk signal was observed in the magnetic data.

### Brain slice preparation

Adult mice (5–10 weeks) were anaesthetised with isoflurane, decapitated and brains were rapidly dissected submerged in ice-cold, carbogen (95% O$_2$/5% CO$_2$) saturated sucrose substituted artificial cerebrospinal fluid (ACSF) containing (in mM): 200 Sucrose, 11 Glucose, 25 NaHCO$_3$, 2.5 KCl, 0.5 L-ascorbic acid, 2 Na-pyruvate, 3 myo-inositol, 1.25 NaH$_2$PO$_4$, 0.1 CaCl$_2$, 4 MgCl$_2$. 400 $\mu$m thick coronal slices containing the *corpus callosum* were obtained using a Leica VT1200s vibratome (Leica Biosystems, Germany). Slices were transferred to an interface holding chamber filled with carbogen saturated regular ACSF containing (in mM): 111 NaCl, 11 Glucose, 25 NaHCO$_3$, 3 KCl, 1.1 KH$_2$PO$_4$, 2.5 CaCl$_2$, 1.3 MgCl$_2$ and allowed to recover at 28C for at least 1 h. The slices were introduced into the same carbogenated ACSF solution, continuously circulated from a reservoir into the solution bath. We chilled the inflow of the ACSF solution into the bath by passing the feed line through an ice bath. This helped dissipate heat from the diamond and AlN heatsink plate, allowing us to maintain the region directly above the sensor at a stable 25C for up to 24 continuous hours. The slice was held down onto the sensor using nylon strings on a nonmagnetic Pt harp structure, ensuring fixed close proximity to the diamond. Stimulation of activity was achieved using current pulses of between 0.2–0.8mA delivered by a PC-triggered, battery powered current source (World Precision Instruments) via a Pt/Ir electrode inserted using micromanipulators into the target region. Stimulation current was adjusted in strength and polarity until a clear characteristic evoked response from the corpus callosum of 0.5-1mV was observed on the electric recording channel.

Our method is not limited to the corpus callosum and allows precision placement of any brain region within the sensing area. Due to the small size of the corpus callosum and the sensing region, we used top-down white light microscopy with zoomable magnification from 2-20 times to locate the correct brain region and position it over the most sensitive point while recording video through a digital camera (IDS) at 60 frames-per-second. Alignment could not be performed by eye. The point of maximum sensitivity was determined prior to slice introduction by imaging the diamond using the top-down white light microscope with the foil and Kapton removed, in order to determine where the pump laser spot illuminated the NV layer (defining the NV sensing volume, see Methods). This position was then marked on the video monitor. To confirm the position directly above the laser spot was the most sensitive, we introduced a fine copper wire to which a 22Hz current signal was applied and which could be driven using a X,Y optomechanical positioner stage (Thorlabs) across the diamond surface with $\approx$5$\mu$m precision. This signal was strongest in the magnetometer readout just above the laser spot both with and without the Kapton/foil layer. Alignment of the brain region and the sensing point could be done with a precision of a few 10s of micrometers using the microscope, carefully positioning the slice using pipette suction.

### Data processing

Measurements were performed without magnetic shielding in an ordinary laboratory (basement) environment. We removed background magnetic noise from electrical mains (primarily 50,150Hz) and non-stationary sources (nearby heating and water pumps) by frequency domain notch filtering, using methodology outlined in detail in our previous works^37,38^. Filtering was performed in a three stage process. First, to avoid ringing, the artifact from the fast stimulation spike (50 $\mu$s) was fitted in the time domain and subtracted (full methodology detailed in Supplementary Information). Secondly, the data was fast Fourier transformed to the frequency domain and the magnetic noise at e.g. 50/150Hz was removed by notch filters. As our signal SNR$<<$1 for a single 60sec time trace, the notch frequencies were identified by a threshold factor of 2-3$\times$ above the white noise floor. A lowpass filter with upper cutoff at 2.5 kHz was implemented to restrict the sensing bandwidth to the frequency range of the target biosignal. In the third and final stage, the signal was inverse Fourier transformed back to the time domain. Epochs of 100 ms, centered on the stimulation triggers were extracted from the filtered data, for a total of 120 in each 60sec acquisition. These epochs were extracted and averaged to reveal the desired biological signal.

### Ethics

All methods in this work were carried out in compliance with the ARRIVE guidelines according to relevant Danish national guidelines and regulations. Experimental protocols were approved where required by the Technical University of Denmark, the University of Copenhagen and the Danish National Committee on Health Research Ethics (DNVK). All methods were carried out in accordance with relevant guidelines and regulations.

## Supplementary Information

Supplementary Information.

## Data Availability

The data that supports the findings of this study are available from the corresponding author upon reasonable request.
