# Supplementary material for: Microscopic-scale magnetic recording of brain neuronal electrical activity using a diamond quantum sensor
Source: Sci Rep. 2023 Jul 31;13:12407. doi: 10.1038/s41598-023-39539-y (PMC10390498; doi:10.1038/s41598-023-39539-y)
Supplement: Supplementary file 1 — Supplementary Information. [file 41598_2023_39539_MOESM1_ESM.pdf]

Microscopic-scale magnetic recording of brain neuronal electrical activity using a diamond quantum sensor

Nikolaj Winther Hansen[1,*], James Luke Webb[2,*], Luca Troise[2,*], Christoffer Olsson[3], Leo Tomasevic[4], Ovidiu Brinza[5], Jocelyn Achard[5], Robert Staacke[6], Michael Kieschnick[6], Jan Meijer[6], Axel Thielscher[3,4], Hartwig Roman Siebner[4,5,6], Kirstine Berg-Sørensen[3], Jean-François Perrier[1], Alexander Huck [2], and Ulrik Lund Andersen[2]

[1] *Department of Neuroscience, University of Copenhagen, 2200 Copenhagen, Denmark*

[2] *Center for Macroscopic Quantum States (bigQ), Department of Physics, Technical University of Denmark, 2800 Kgs. Lyngby, Denmark*

[3] *Department of Health Technology, Technical University of Denmark, 2800 Kgs. Lyngby, Denmark*

[4] *Danish Research Center for Magnetic Resonance, Center for Functional and Diagnostic Imaging and Research, Copenhagen University Hospital - Amager and Hvidovre, 2650 Hvidovre, Denmark*

[5] *Laboratoire des Sciences des Procédés et des Matériaux, Université Sorbonne Paris Nord, 93430 Villetaneuse, France*

[6] *Division Applied Quantum System, Felix Bloch Institute for Solid State Physics, Leipzig University, 04103, Leipzig, Germany*

[5] *Department of Neurology, Copenhagen University Hospital Bispebjerg and Frederiksberg, 2400 Copenhagen, Denmark*

[6] *Department of Clinical Medicine, Faculty of Health and Medical Sciences, University of Copenhagen, 2200 Copenhagen N, Denmark*

[*] *These authors contributed equally to this work*

1 Sensor Sensitivity

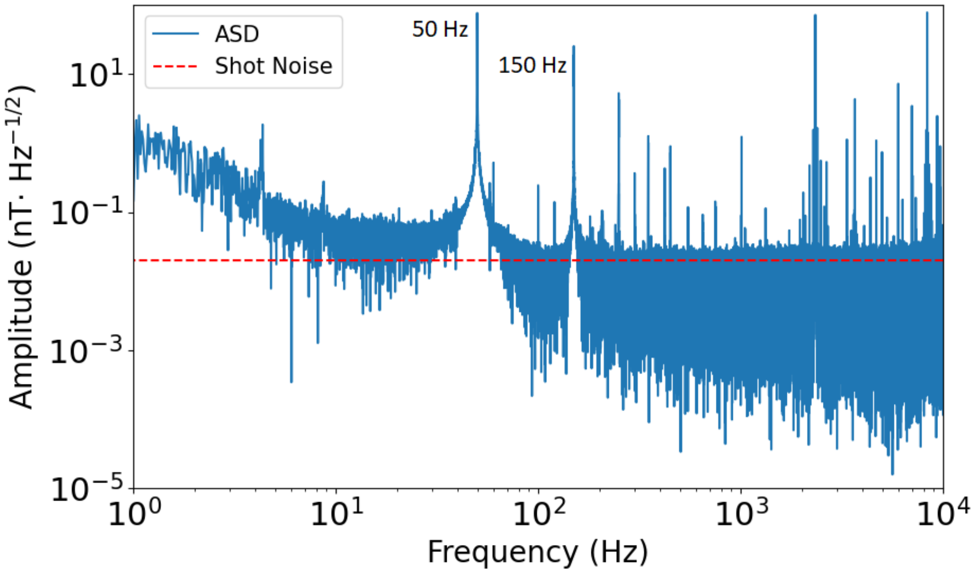

Supplementary Figure 1: Amplitude spectral density (ASD) plot, detailing the sensitivity of the quantum sensor in nT/$\sqrt{Hz}$ as a function of magnetic signal frequency. The ASD is an example, plotted for a single 60sec acquisition. A floor of approximately 50-70pT/$\sqrt{Hz}$ was reached.

Supplementary Figure 1 details the sensitivity of our quantum sensor in terms of the amplitude spectral density (in units of nT/$\sqrt{Hz}$) as a function of target magnetic field signal frequency. For a single 60sec acquisition, a floor of approximately 50-70pT/$\sqrt{Hz}$ was reached with an acquisition bandwidth of approximately 10kHz, limited by lock-in amplifier low pass frequency (time constant). Based on the collected fluorescence power (5-6mW), we estimated the shot noise limited sensitivity to be $\approx$16nT$\sqrt{Hz}$. We plot this level as the dashed red line on the above figure, assuming a flat white noise spectrum. The gap between the shot noise level and the measured sensitivity we attribute to imperfect rejection of common mode (predominantly laser technical) noise by our balanced detector, as well as magnetic noise from the laboratory background. Here the main source is from electrical mains (50Hz) and inductive transformer harmonics (mainly at 150Hz). The frequency distribution of the mains noise is broadened by mains phase drift. Based on this measured noise level, our single shot magnetic field measurement noise without any removal of background magnetic noise is approximately 2μT using a sensing bandwidth of 10kHz and 150nT when constraining bandwidth to 2.5kHz by short pass filtering. By implementing de-noising in post-processing, this can be reduced to 7nT and 3nT for 10kHz and 2.5kHz respectively. This is only possi-

ble thanks to the large dynamic range of the NV sensor. Alternative sensing platforms (in particular those based on atomic vapour) can be saturated by the high level of background magnetic noise.

2 Control Slice: No Tetrodotoxin

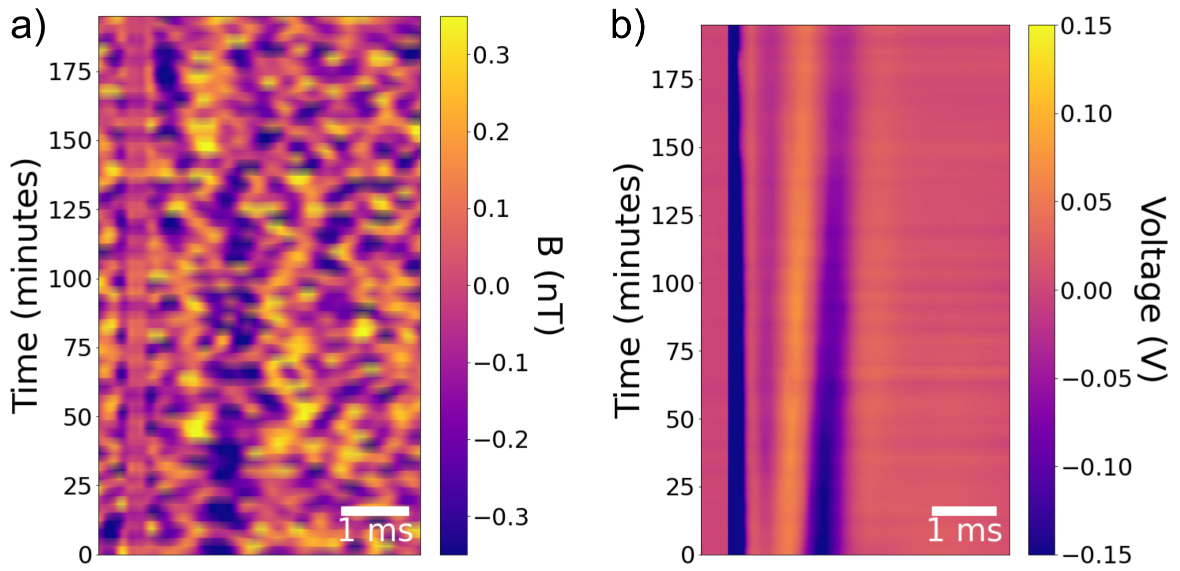

Supplementary Figure 2: Control measurements for a brain slice without the addition of TTX. Magnetic field data from the sensor is shown in a) and reference electrical data in b).

Supplementary Figure 2 shows the biological signal as a function of time with zero tetrodotoxin (TTX) added to the solution bath. Data is shown for the first 3 hours of recording. The signal was observed to slowly decay in amplitude over time as the slice slowly died, with increased latency of the signal component S2.

3 Minimisation of Stimulation Artifact

As typically observed in electrophysiology experiments, electrical tissue stimulation also induces a stimulation artifact resulting from stimulation current propagation in the tissue and solution bath. This was also observed in the magnetic data from our quantum sensor. Such an artifact poses a problem for digital filtering, the sharp pulse of the stimulation artifact inducing ringing artifacts effects in the filtered data. This is exemplified in Supplementary Figures 4,a) and b), where a 3rd order Butterworth low-pass filter with cutoff frequency

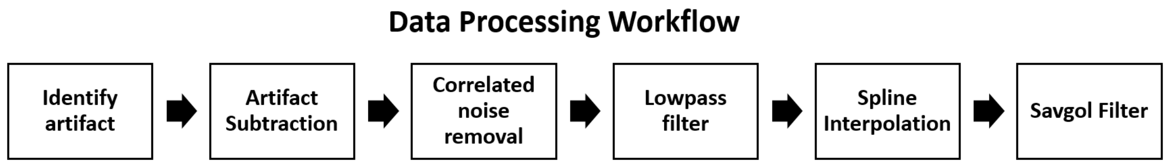

Supplementary Figure 3: Data processing workflow of the artifact removal process.

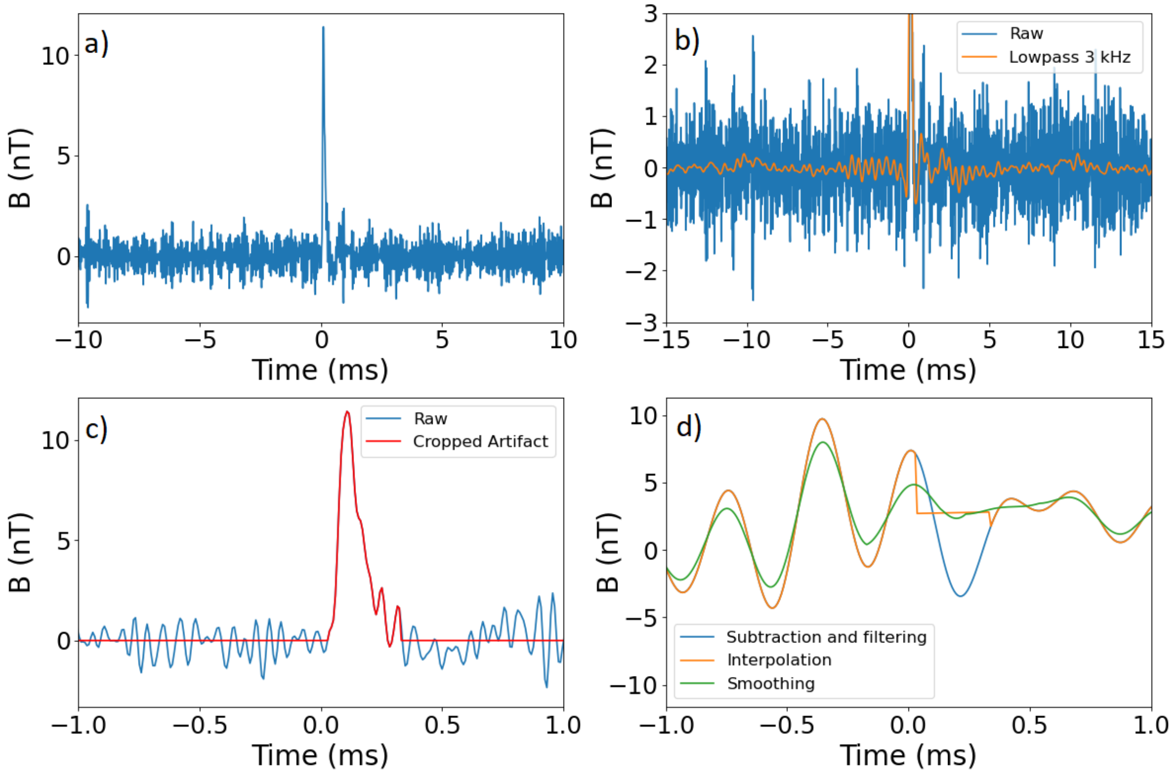

Supplementary Figure 4: Example of an averaged stimulation spike in the magnetic data. The amplitude of such spike is one order of magnitude larger then the biological response from the corpus callosum. b) Comparison of the same stimulation spike shown in a) before and after applying a lowpass filter with a 3 kHz cutoff frequency. As a result of the filtering, ringing artifacts are generated before and after the spike. c) Cropped stimulation spike used for subtraction. d) Comparison of the same single-shot time trace after applying spike subtraction and low-pass filter (blue), interpolation (orange) and smoothing (green).

at 3 kHz was applied to an example stimulation event. The unmistakable oscillating ringing artifacts caused by the filtering are visible, with amplitudes in the order of hundreds of picotesla.

In order to tackle this problem, we therefore post-processed our magnetic data to remove stimulation artifacts in a process detailed in Supplementary Figures 3 and 4,c) and d). In

brief, the average signal spike caused by the stimulations was first identified in the time domain and and subtracted from each individual stimulation event in the magnetic data, before applying the majority of signal filtering in the frequency domain. Any residual artifact remaining was then removed by interpolation in the time domain. This approach is a standard, well established method in the electrophysiology literature for stimulation artifact removal. However, here we face unique challenges: in particular the presence of a high level of magnetic background noise and far lower signal level in our magnetic data. This required a modified method for artifact removal.

Identification of the stimulation artifact was carried out by attenuating background mains noise using digital notch filters at 50, 150 and 250 Hz, followed by averaging 60 time traces of 60s at time, for a total of 7000 stimulations. This was necessary to reduce the background mains noise below the artifact level (several nT) while retaining sensing bandwidth (10 kHz). Using this averaged data, we then defined the duration of the artifact as the interval between the stimulation trigger (at t=0sec) and the second zero-crossing of the signal after the peak (Supplementary Figure 4,c).

The averaged artifact signal present in this interval was then cropped and subtracted from each stimulation event in all the magnetic data averaged for the identification process. We note that the success of this procedure relies on the amplitude and duration of the artifact for each stimulation event to be relatively consistent and close to the mean average stimulation artifact. We found this to be the case in the experiments in this work, with variation in amplitude of less than 10%. After the subtraction, the time traces were filtered in the frequency domain to remove noise (as described in Methods).

As we subtract only the average artifact, we note that the subtraction process leaves a residual stimulation artifact, as a result of the imperfect subtraction from each stimulation event of the averaged response. To remove this residual, the magnetic data was further processed by masking the time intervals containing the artifacts, of mask duration earlier obtained during the peak identification process. The signal between these masked sections was then interpolated using 1st order spline interpolation (Supplementary Figure 4,d), with this interpolated data replacing the masked artifact sections in the magnetic data. Finally, a Savitskzy-Golay filter was then applied to each 60s timeseries of this processed magnetic data to smooth the edges of the interpolation intervals.

For completeness, in Supplementary Figures 6-8 we show that the biological signal can be observed with minimal postprocessing and filtering and without artifact removal, employing only a lowpass filter at 6kHz and notch filters at mains harmonics (50,150,250Hz). This removes the majority of the background magnetic noise which obscures the signal (see Sup-

plementary Figure 5). The ringing artifacts as a result of retaining the stimulation artifact can be seen, partially obscuring the biological signal.

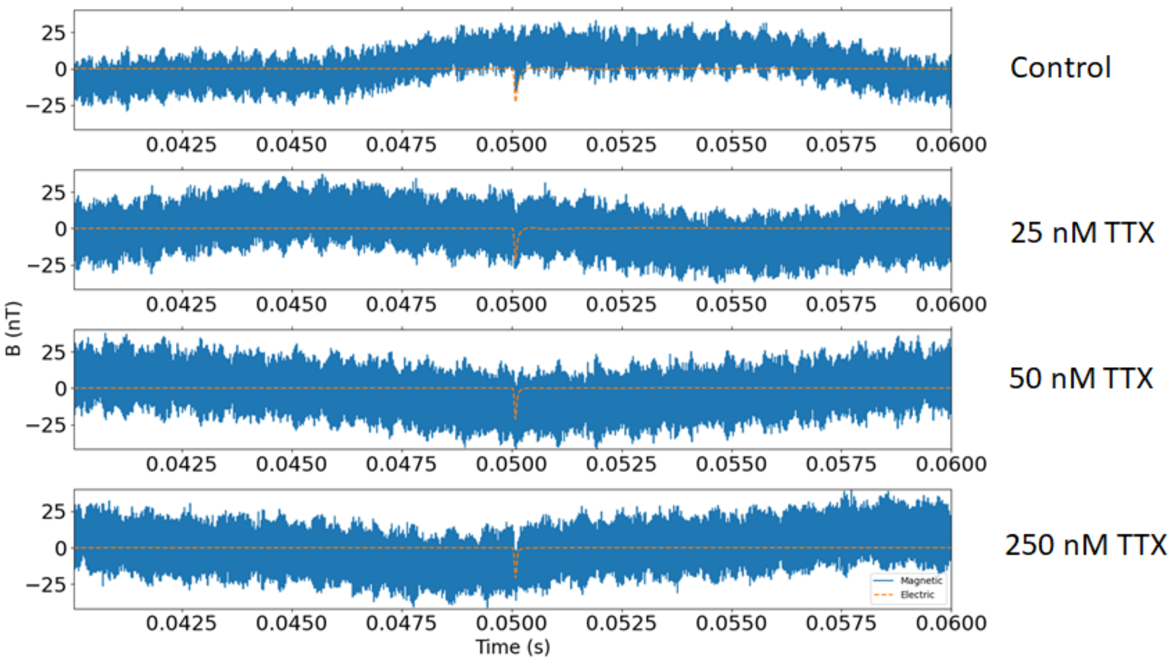

Supplementary Figure 5: Data for Slice 1 with no filtering or stimulation artifact removal on either the electric or the magnetic data, only averaging for each concentration of TTX. For the magnetic data, any biological signal is entirely obscured by background magnetic noise, primarily at 50Hz and higher mains harmonics. Electrical capacitive pickup of 50Hz on the electrical probe readout also eliminates the biological signal, leaving only a residual stimulation artifact.

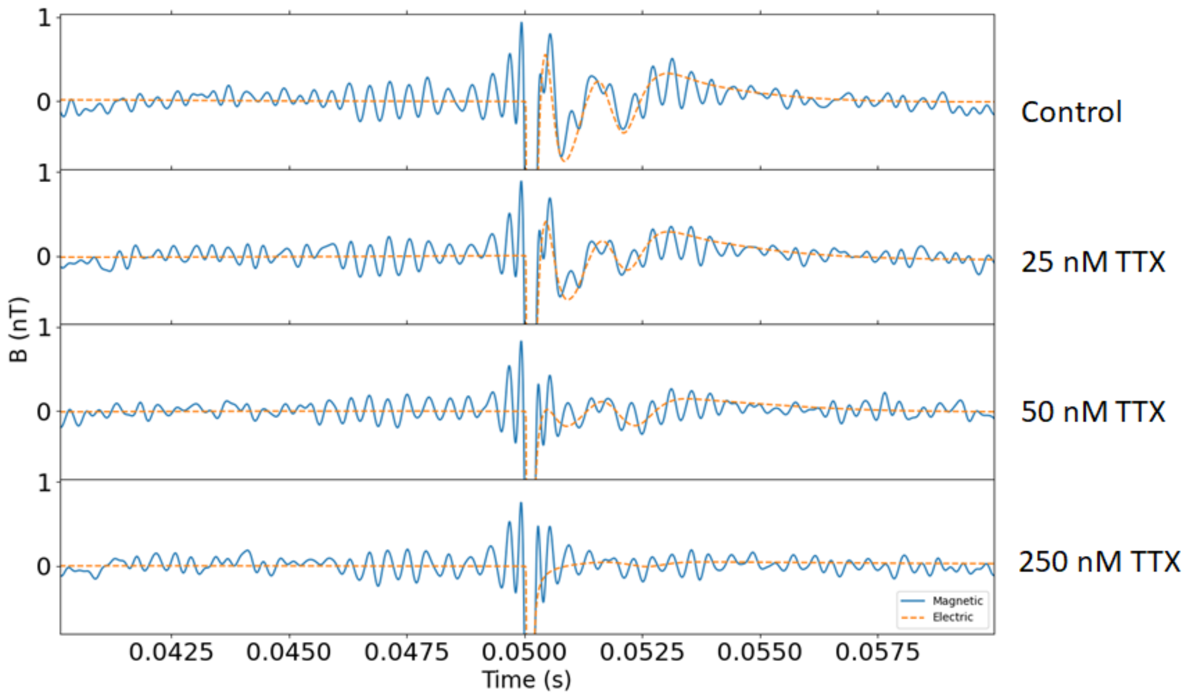

Supplementary Figure 6: Data for Slice 1 with minimal filtering and no stimulation artifact removal (6kHz low pass filter and mains notch filters at 50,150,250Hz). The biological signal is visible in the magnetic data, but are partially obscured by the ringing effects as a result of the stimulation artifact. Removal of the artifact by the above procedure avoids these issues.

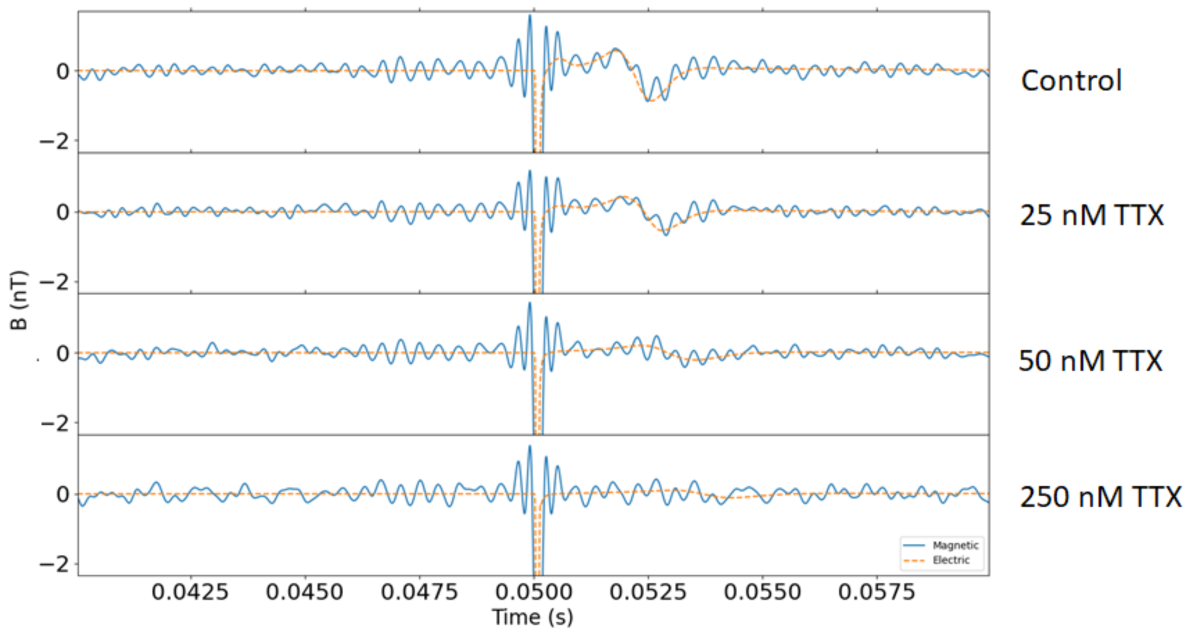

Supplementary Figure 7: Data for Slice 3 with minimal filtering and no stimulation artifact removal (6kHz low pass filter and mains notch filters at 50,150,250Hz)

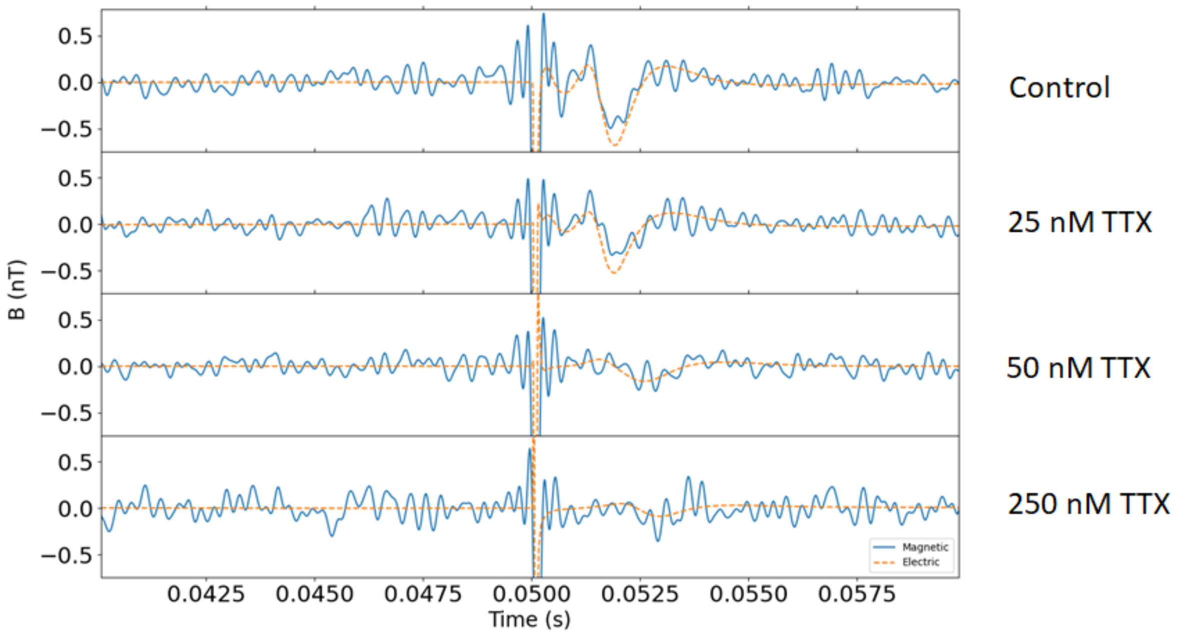

Supplementary Figure 8: Data for Slice 2 with minimal filtering and no stimulation artifact removal (6kHz low pass filter and mains notch filters at 50,150,250Hz)

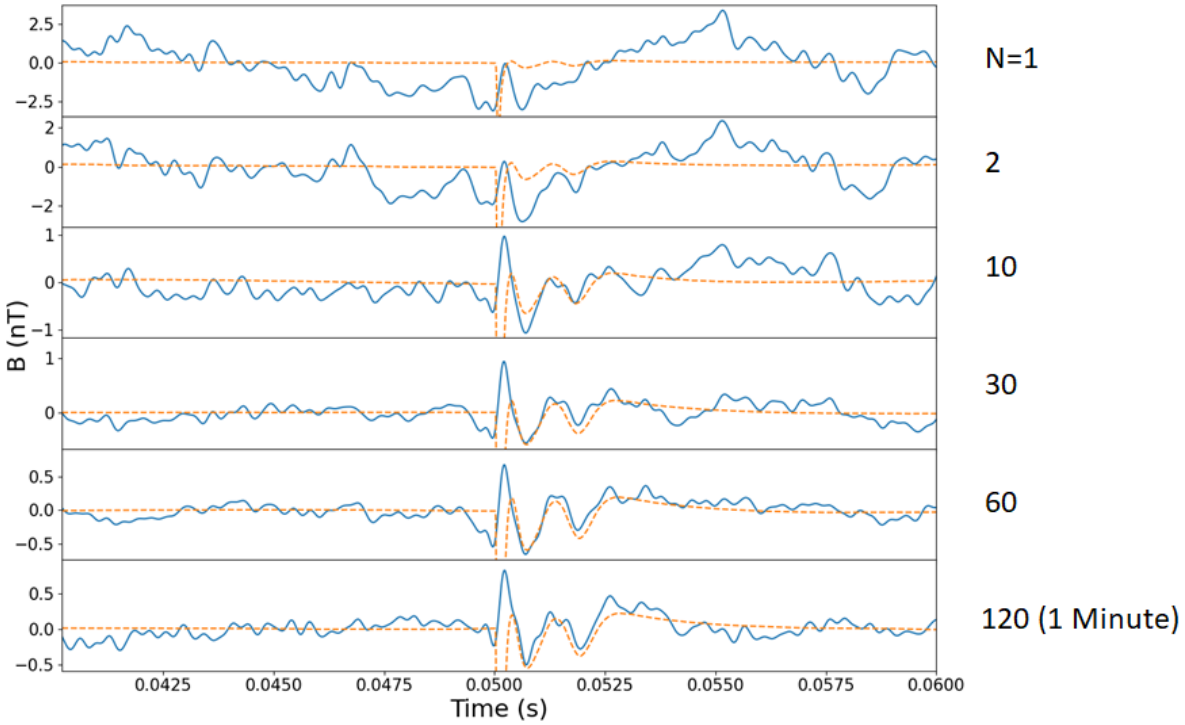

Supplementary Figure 9: Magnetic signal as a function of number of stimulations averaged, from single shot (N=1) to N=120. Both parts of the biological signal are visible after approximately N=20 stimulations.

4 ODMR Spectra

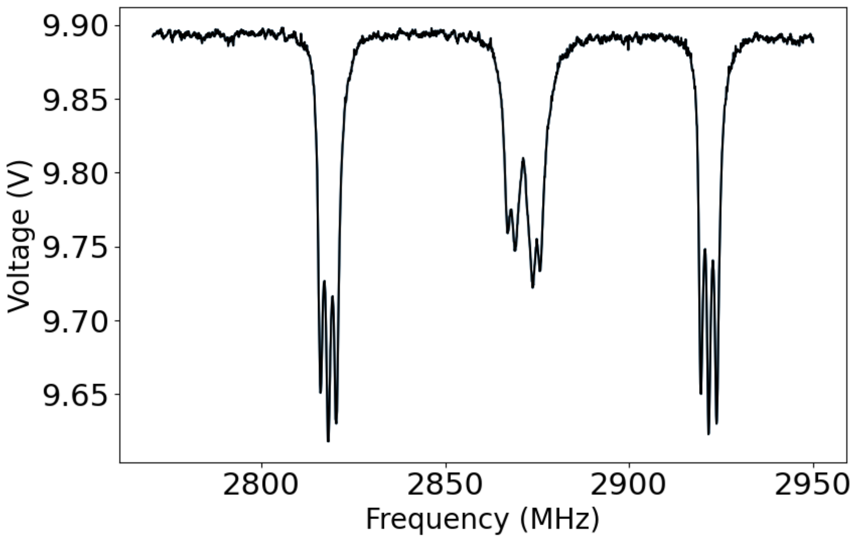

Supplementary Figure 10: Example of experimental ODMR spectra as DC photodetector voltage

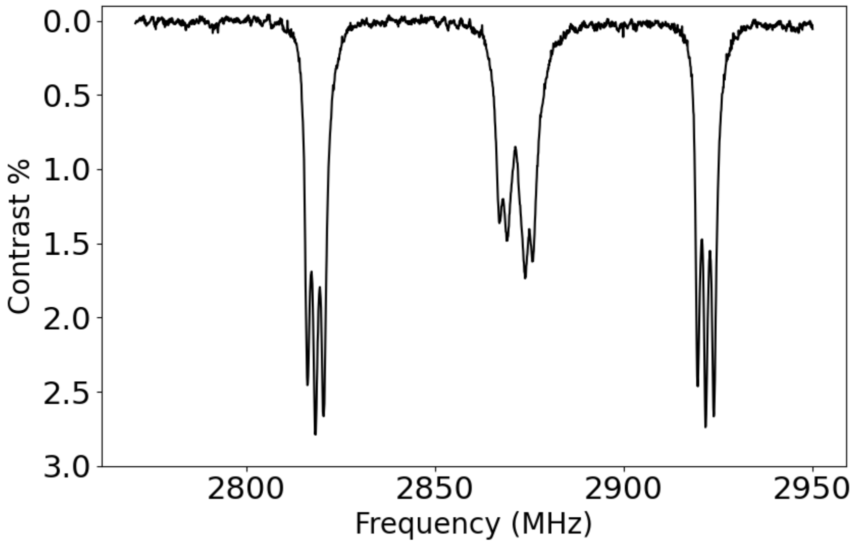

Supplementary Figure 11: Example of experimental ODMR spectra converted to units of contrast, defined as the percentage change from off to on microwave resonance with the NV spin and hyperfine-split energy levels.

Supplementary Figures 10, 11 and 12 show examples of the optically detected magnetic resonance (ODMR) spectra from the experiment. Supplementary Figures 10 and 11 show the same ODMR data recorded in terms of photodetector voltage (linearly proportional to

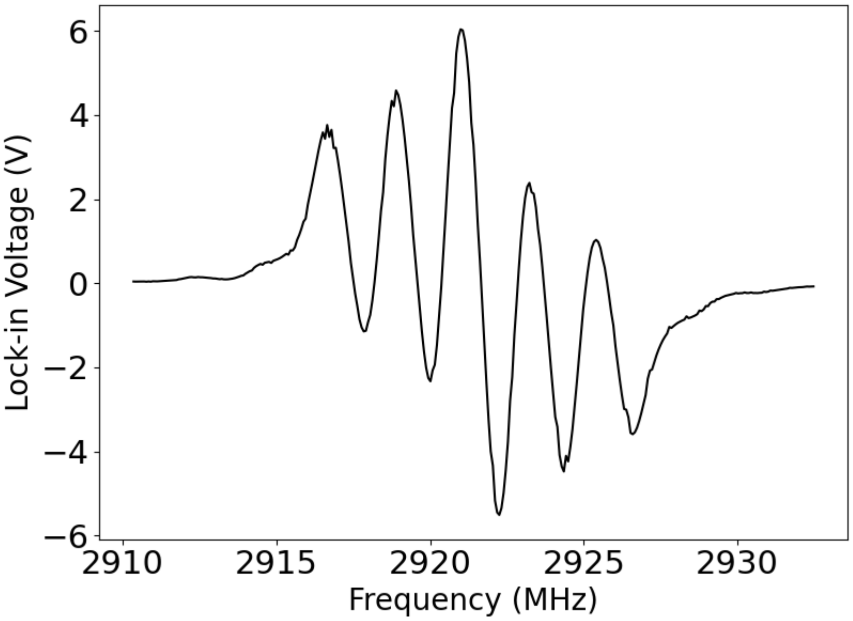

Supplementary Figure 12: Example of experimental ODMR spectra using 3-frequency drive, in units of absolute (R) lock-in amplifier output, set at an amplification level of input 500mV equal to full scale 10V. A greater relative change in output voltage as a function of frequency yields an improved magnetic field sensitivity for the same noise level.

collected fluorescence power) and converted to units of ODMR contrast. Measurements were performed using the same balanced detector, but switched to a mode that recorded only the fluorescence signal collection with no reference subtraction.

Supplementary Figure 12 shows an example of ODMR measured via lock-in amplifier demodulation. Here the 2.925GHz microwave carrier was frequency modulated at 33.3kHz and mixed with an additional 2.16 MHz signal to drive all 3 nitrogen-14 hyperfine transitions. As per El-Ella et al. (see reference in main text), this 3-frequency drive method yields a greater response to magnetic field - in terms of ODMR spectrum slope - than using a single drive.
